# Proactive pharmacogenomics in azathioprine-treated pediatric inflammatory bowel disease at a Chinese tertiary hospital

**DOI:** 10.3389/fphar.2025.1558897

**Published:** 2025-03-26

**Authors:** Cai-Yun Long, Ying Huang

**Affiliations:** Department of Gastroenterology, Children’s Hospital of Fudan University, National Children’s Medical Center, Shanghai, China

**Keywords:** pediatric inflammatory bowel disease, azathioprine, pharmacogenomics, myelosuppression, TPMT, NUDT15

## Abstract

**Background:**

Despite the emergence of numerous innovative targeted therapies for the management of pediatric inflammatory bowel disease (IBD), azathioprine continues to be a pivotal first-line therapeutic agent. Nonetheless, the considerable frequency of myelosuppression associated with its use warrants careful consideration and further investigation. This study aims to investigate the application of pharmacogenomics in Chinese pediatric IBD treated with azathioprine, and to elucidate its association with the occurrence of myelosuppression.

**Methods:**

We conducted a retrospective analysis to determine the prevalence of pharmacogenetic abnormalities and thiopurine-induced myelosuppression in Chinese pediatric patients with IBD.

**Results:**

Among the 227 patients underwent pharmacogenetic testing, abnormal genetypes occurred in 66 patients, among which 7 patients exhibited aberrant *TPMT* and 59 had aberrant *NUDT15*. Of the 58 patients who were treated with azathioprine, 23 cases experienced myelosuppression. All three children with heterozygous mutations in *NUDT15* developed leukopenia following azathioprine treatment. Among patients with normal pharmacogenetic results, 20 cases (36.4%) developed myelosuppression, while 35 cases (63.6%) did not. The dose of azathioprine was below the recommended level in guidelines. The mean dose of azathioprine (mg/kg/day) in the myelosuppression group was 1.22 ± 0.32, compared to 1.42 ± 0.42 in the non-myelosuppression group, which represented a statistically significant difference (p < 0.05). Age, gender, and the use of concomitant biologics, mesalazine, or glucocorticoids did not show significant differences between the groups (p > 0.05).

**Conclusion:**

*NUDT15* C415T is prevalent in China and is associated with an increased risk of azathioprine-induced myelosuppression. A reduced dose of azathioprine should be considered for Chinese pediatric patients with IBD, even in those with normal pharmacogenetic profiles.

## 1 Introduction

Thiopurines, such as 6-mercaptopurine, 6-thioguanine, and azathioprine, are extensively employed in the management of acute lymphoblastic leukemia and autoimmune conditions, including inflammatory bowel disease (IBD) and rheumatoid arthritis. The adverse effects associated with these drugs are notable and warrant attention. Up to 10%–30% of patients experience treatment intolerance due to side effects, which may entail myelosuppression, hepatotoxicity, pancreatitis, and nausea. Myelosuppression is a frequent severe adverse event, with an incidence ranging from 3% to 7%, and it can result in life-threatening infections (Ribeiro et al., 2023). Therefore, monitoring for treatment-related myelosuppression in patients with inflammatory bowel disease is imperative.

The metabolism of thiopurines is catalyzed by two principal detoxifying enzymes, TPMT and NUDT15, which serve as negative regulators of thiopurine activity and toxicity. Variants within the *TPMT* and *NUDT15* alleles exhibit diverse frequencies across the general population, resulting in considerable interindividual variability in pharmacological and toxicological responses to thiopurine-based medications. Mutations in these enzymes are known to increase the risk of myelosuppression in patients (Kim et al., 2010; Banerjee et al., 2020; Gutiérrez-Valencia et al., 2023). Consequently, the implementation of pharmacogenomic testing for *TPMT* and *NUDT15* can effectively pinpoint individuals at higher risk for adverse events associated with thiopurines (Van Den Bosch and Coenen, 2021; Dickson et al., 2022; Pratt et al., 2022). However, the distribution of genetic mutations varies across different regions and ethnic groups, and the disease itself may affect the absorption and metabolism of drugs. Therefore, it is necessary to explore pharmacogenomics in populations with particular diseases in certain regions.

Pediatric IBD exhibits unique features distinct from adult IBD. Nevertheless, to date, research on pharmacogenomic testing for azathioprine and its specific clinical implications for pediatric IBD patients, particularly in the context of East Asia, has been limited (Kennedy et al., 2024). This study conducted a retrospective analysis of the pharmacogenomic data for azathioprine in Chinese pediatric patients with IBD, summarized the prevalence of various genotypes and assessed the incidence of adverse reactions following azathioprine use, aiming to provide insights that could inform clinical practice in the management of pediatric IBD in China.

## 2 Materials and methods

### 2.1 Ethics and informed consent

This study was conducted in accordance with the Declaration of Helsinki and was approved by the Institutional Review Board of our institution. Informed consent was obtained from the legal guardians of participants.

### 2.2 Inclusion criteria for participants

The inclusion criteria for the study were as follows.

• Children diagnosed with IBD at our hospital between July 2019 and May 2024.
• Patients who had entered the maintenance treatment phase.
• Peripheral white blood cell counting (WBC) > 4.0 * 10^9^/L, hemoglobin (HGB) > 100 g/L, platelet (PLT) > 100 * 10^9^/L
• Patients who had undergone *TPMT* and *NUDT15* genetic testing.

### 2.3 Genotyping analysis

Venous blood samples of 2 mL were collected, anticoagulated with EDTA, and then sent to Shanghai Jinyu Medical Testing Co., Ltd. For azathioprine pharmacogenetic analysis. Briefly, DNA was extracted from blood samples, and after passing quality control, the target fragments were amplified and libraries were constructed, followed by next-generation sequencing. Based on the joint consensus recommendation of pharmacogenetics, the pharmacogenetic testing covered three *TPMT* loci (TPMT*2 c.238G>C, TPMT*3B c.460G>A, and TPMT*3C.719A>G), as well as one *NUDT15* locus (*NUDT15* c.415 C>T) (Pratt et al., 2022).

### 2.4 Collection of clinical data

The following clinical data were collected for subsequent analyses.

• General information: this includes the participant’s name, gender, age, and weight.
• Time and results of *TPMT* and *NUDT15* genetic testing.
• Hematological parameters including WBC, PLT, and HGB, both before and after the initiation of medication.
• Azathioprine medication details: this encompassed the start time of azathioprine therapy, the initial dose, any subsequent adjustments to the medication’s dosage.
• Concomitant medications such as biologics, mesalazine, and corticosteroids.
• Definition of myelosuppression: patients with peripheral WBC <4 * 10^9^/L, HGB <100 g/L, or PLT <100 * 10^9^/L, were considered with myelosuppression (Nian et al., 2024).

### 2.5 Statistical methods

IBM SPSS 20.0 software was used for the statistical analysis of the data. Continuous variables were reported as mean ± standard deviation. As for statistical comparison between groups, independent *t* was used for continuous variables, while *χ* ^*2*^ was applied for categorical variables. P < 0.05 was defined as statistically significant.

## 3 Results

### 3.1 General information

**FIGURE 1:**
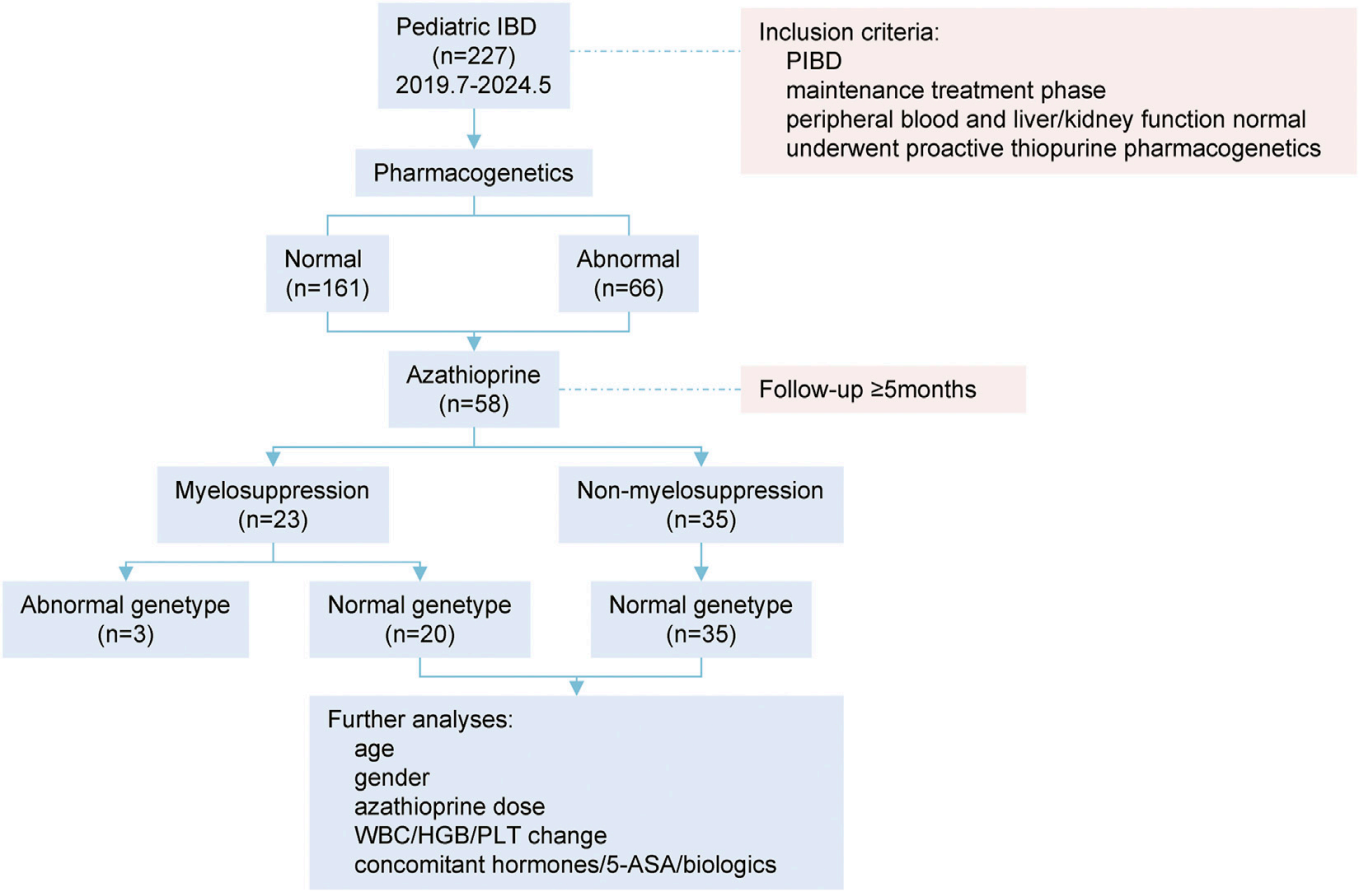
Flow chart of study design. PIBD: pediatric inflammatory bowel disease; WBC: white blood cell counting; HGB: hemoglobin; PLT, platelet; 5-ASA: mesalazine.

Flow chart of study design was illustrated in Figure 1. Between July 2019 and May 2024, 227 children with IBD were subjected to azathioprine pharmacogenetic testing. The age of the children spanned from 1 to 17 years, with a mean age of 10.83 ± 3.74 years. The gender distribution was 143 males and 84 females. The disease subtypes were as follows: 176 cases of Crohn’s disease (CD), 33 cases of ulcerative colitis (UC), and 18 cases of IBD unclassified (Table 1).

**TABLE 1:** Clinical data of patients with proactive pharmacogenomic testing. CD: Crhon’s disease; UC: ulcerative colitis; IBDU: IBD unclassified.

	Number (%)
Total number	227
Gender	
Male	143 (63.0%)
Female	84 (37.0%)
Age (year, mean ± SD)	1–17 (10.83 ± 3.74)
Subtype	
CD	176 (77.5%)
UC	33 (14.5%)
IBDU	18 (8.0%)
Metabolic activity	
Normal	161 (70.9%)
Intermediate	62 (27.3%)
Poor	4 (1.8%)

### 3.2 Pharmacogenetic profiles and metabolic activities

**FIGURE 2:**
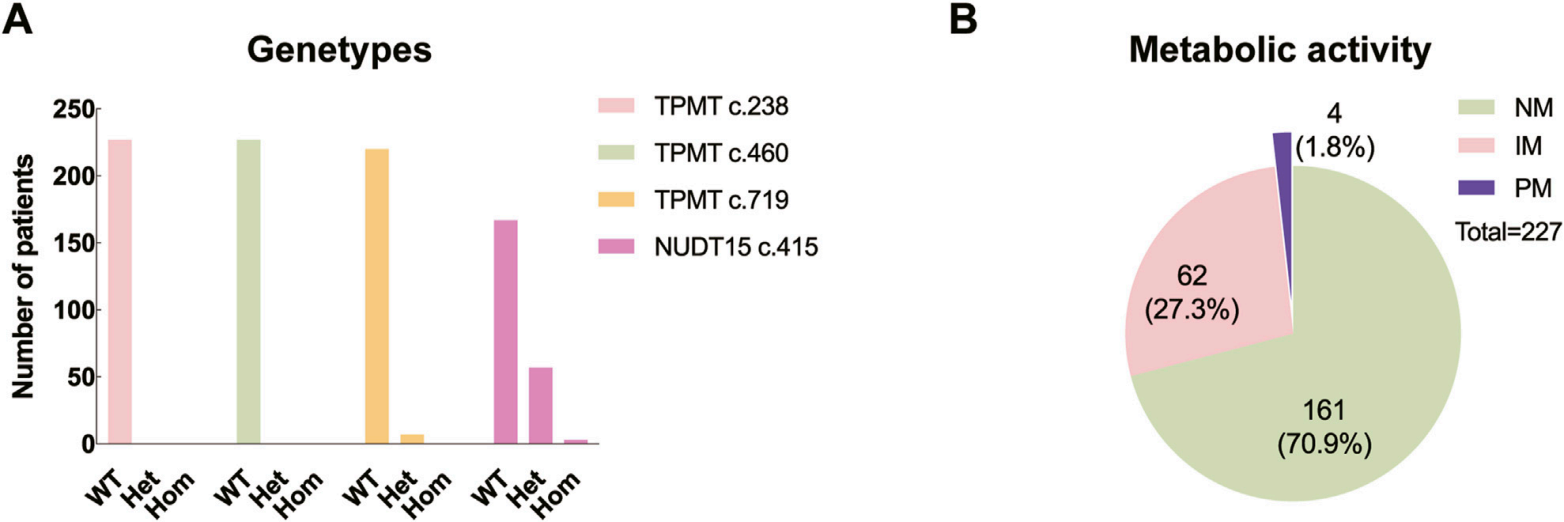
Distribution of genetypes and metabolic levels. **(A)** WT: wild type; Het: heterozygous; Hom: homozygous. **(B)** NM: normal metabolic activity; IM: intermediate metabolic activity; PM: poor metabolic activity.

Among the children, 161 (70.9%) exhibited normal pharmacogenetic results, whereas 66 (29.1%) had abnormal findings. The *TPMT* c.238 and *TPMT* c.460 loci were homozygous wild-type in all cases (100%). As for *TPMT* c.719 locus, the wild-type was present in 220 cases (96.9%), while the wild-type *NUDT15* c.415 was identified in 167 cases (73.6%) (Figure 2A).

Genotype reports detail the genetic polymorphisms, whereas the predicted phenotypes concern how the genotypes influence the activity of the specific proteins. According to the commonly used phenotype nomenclature for thiopurine metabolizing enzymes, “normal metabolizer” means “two normal function alleles (i.e., wild type), “intermediate metabolizer” means “one loss/null function allele and one normal/supra-function allele”, and “poor metabolizer” means “two loss/null function alleles” (Sandritter et al., 2023). In this study, of those with genetic abnormalities, 62 (27.3%) presented with intermediate metabolic activity, including 6 with *TPMT* c.719A/G variant and 56 with *NUDT15* c.415C/T variant; meanwhile, 4 (1.8%) presented with poor metabolic activity, consisting of three patients with the *NUDT15* c.415T/T variant and one patient with both the *TPMT* c.719A/G and *NUDT15* c.415C/T variants (Figure 2B).

### 3.3 The use of azathioprine in diverse pharmacogenetic contexts

Due to significant interethnic differences in thiopurine pharmacogenomics, evidence that lower doses of 6-mercaptopurine/azathioprine achieve sufficient clinical efficacy and therapeutic erythrocyte 6-thioguanine nucleotide (6-TGN) concentrations in Japanese adult IBD patients, and the known differences between pediatric and adult IBD, we adopted a step-up dosing strategy with initial doses below recommended levels (Komiyama et al., 2008; Desai et al., 2023; Gutiérrez-Valencia et al., 2023). In line with guideline recommendations, we closely monitored patients’ complete blood counts (Relling et al., 2019). The dose was gradually increased if no adverse effects were observed, or reduced/discontinued if adverse effects occurred.

In this study, a total of 58 patients who were receiving azathioprine therapy and had completed a follow-up period of ≥5 months were included for further analyses. Of these patients, 55 cases had normal pharmacogenetic results, and 3 cases had abnormal results, all of which were due to the *NUDT15* c.415C>T variant. Patient 1, a 12-year-old male with CD, was treated with azathioprine at 1.32 mg/kg/d and corticosteroids; after 1 month, his white blood cell count decreased from 4.45 to 3.87 (*10^9^/L). Patient 2, an 11-year-old male with CD, was treated with azathioprine at 1.36 mg/kg/d, adalimumab, and thalidomide; after 2 months, his white blood cell count decreased from 5.1 to 3.38 (*10^9^/L). Patient 3, a 7-year-old female with indeterminate IBD, was treated with azathioprine at 0.74 mg/kg/d and mesalazine; after 1.5 months, her white blood cell count decreased from 5.7 to 2.41 (*10^9^/L). Regarding drug-related adverse reactions, only Patient one experienced an increase in transaminase levels during the treatment.

Overall, the initial dose of azathioprine for these patients was 0.29–1.61 mg/kg/day (0.79 ± 0.28), and the maximum dose was 0.70–2.50 mg/kg/day (1.29 ± 0.37). The adverse reactions included increased transaminases in 4 cases, gastrointestinal discomfort in 7 cases, rash in 9 cases, influenza-like symptoms in 5 cases, pancreatitis in 1 case, and myelosuppression in 23 cases (Table 2).

**TABLE 2:** Adverse reactions after using azathioprine in patients with various pharmacogenetic genotypes.

	TPMT *2	TPMT *3B	TPMT *3C		NUDT15 C415T		
	G/G	G/G	A/A	A/G	C/C	C/T	T/T
Number	227	227	220	7	167	57	3
Use azathioprine	58	58	57	1	55	3	0
Adverse events							
Aminotransferase elevation	4	4	4	0	3	1	-
Gastrointestinal discomfort	7	7	7	0	7	0	-
Rash	9	9	9	0	9	0	-
Influenza-like symptoms	5	5	5	0	5	0	-
Pancreatitis	1	1	1	0	1	0	-
Myelosuppression	23	23	23	0	20	3	-

### 3.4 Myelosuppression after azathioprine administration

**FIGURE 3:**
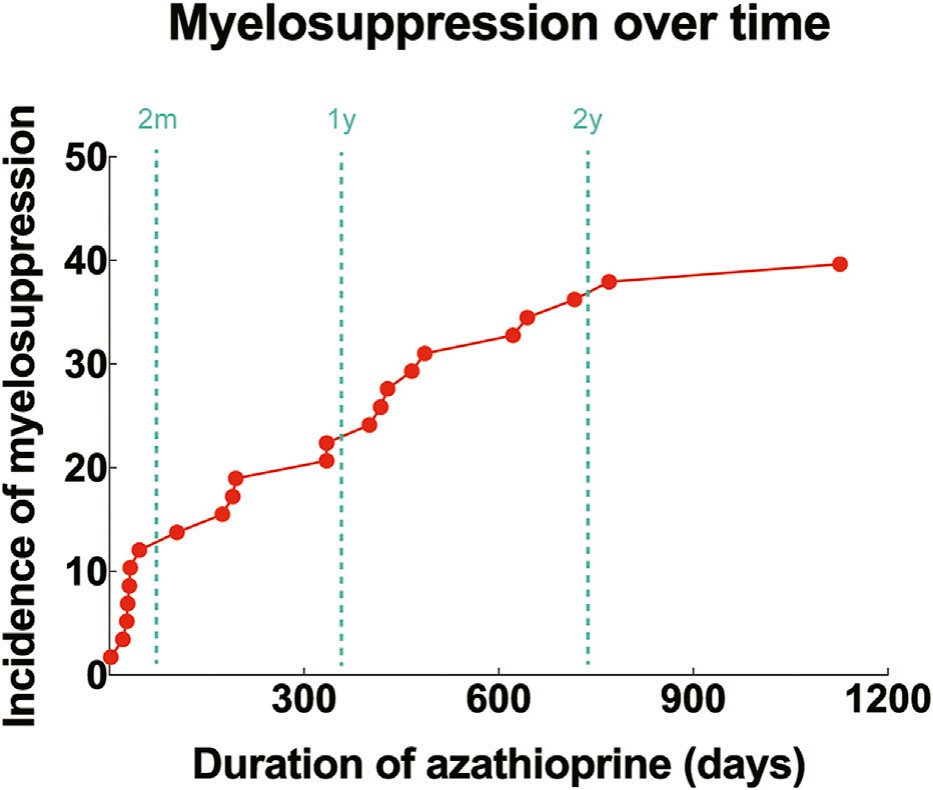
Incidence of myelosuppression over time after initiation of azathioprine. Red dots: cumulative incidence of myelosuppression corresponding to different durations of drug use (%); dashed lines: time points of 2 months, 1 year and 2 years, respectively.

Among the 58 children treated with azathioprine, 23 experienced myelosuppression, while 35 did not. Three patients with genetic abnormalities all developed myelosuppression following azathioprine treatment (100.0%). The onset of myelosuppression ranged from 2 to 1,126 days (mean ± SD: 300.3 ± 330.4) after the initiation of therapy. Seven cases (30.4%) developed myelosuppression within 60 days of starting medication, and 16 cases (69.6%) developed it after 60 days (Figure 3). Of the three children with genetic abnormalities, two experienced myelosuppression within 60 days (31 and 46 days, respectively), and one experienced it after 770 days of medication.

In the myelosuppression group, the starting dose and maximum dose of azathioprine were 0.29–1.32 mg/kg/day (0.79 ± 0.28) and 0.74–1.96 mg/kg/day (1.34 ± 0.34), respectively. The average decreases in WBC, HGB, and PLT after azathioprine administration were 3.21 * 10^9^/L, 0.06 g/L, and 38.44 * 10^9^/L, respectively. In the non-myelosuppression group, the starting dose and maximum dose of azathioprine were 0.43–1.61 mg/kg/day (0.80 ± 0.29) and 0.70–1.81 mg/kg/day (1.22 ± 0.32), respectively. The average decreases in WBC, HGB, and PLT were 2.85 * 10^9^/L, 1.43 g/L, and 32.43 * 10^9^/L, respectively.

**FIGURE 4:**
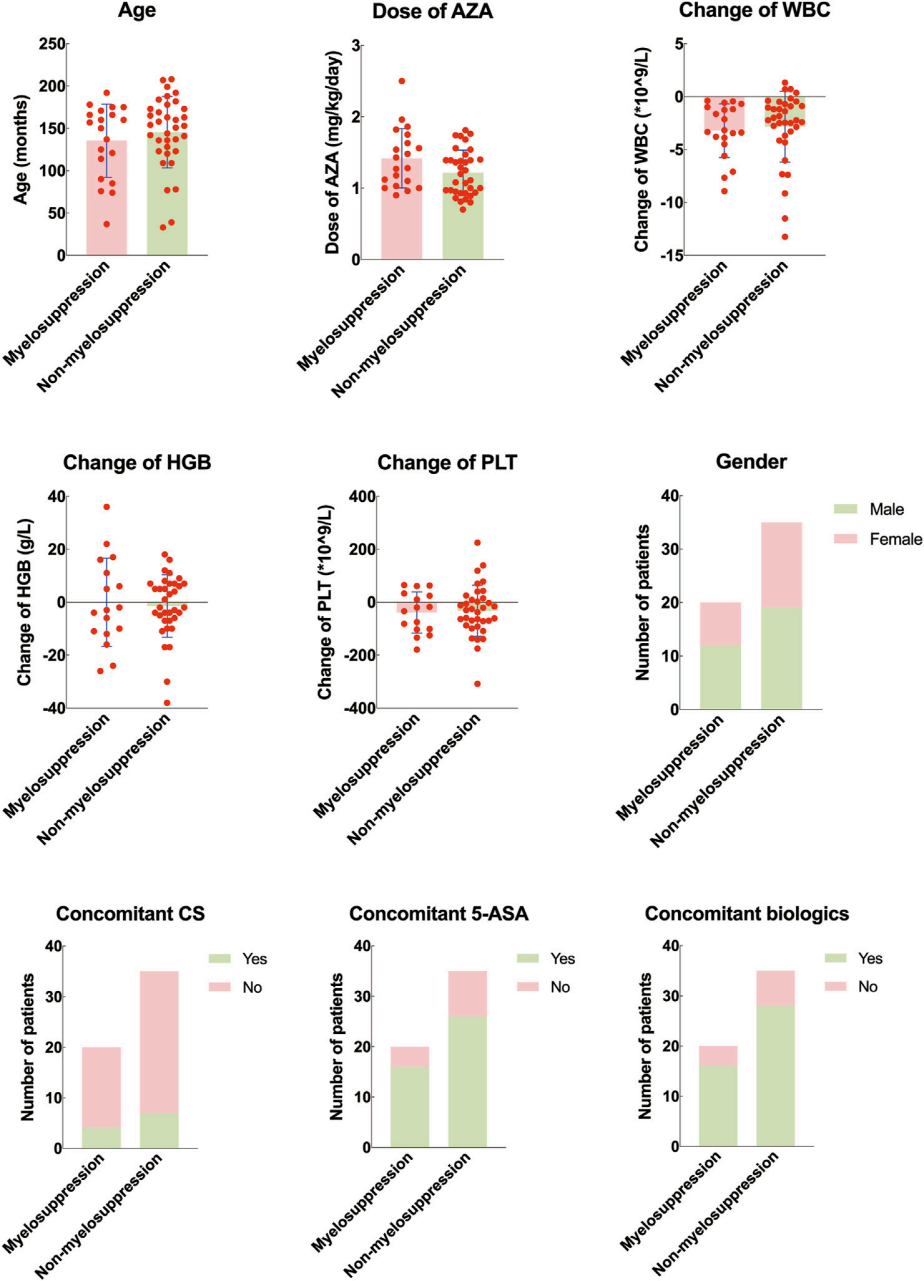
Difference of clinical features between pediatric inflammatory bowel disease with and without myelosuppression. AZA, azathioprine. WBC: white blood cell counting; HGB: hemoglobin; PLT: platelet; 5-ASA: mesalazine.

There was a statistically significant difference in the maximum dose of azathioprine between the myelosuppression group and the non-myelosuppression group (P < 0.05). No statistically significant differences were found in age, gender, starting dose of azathioprine, and concomitant use of hormones, mesalazine, or biologics between the two groups (P > 0.05) (Table 3; Figure 4).

**TABLE 3:** Myelosuppression and other clinical characteristics in children treated with azathioprine. AZA, azathioprine. WBC: white blood cell counting; HGB: hemoglobin; PLT, platelet.

	Myelosuppression	Non-myelosuppression	P
Number	23	35	
Age (year, mean ± SD)	3–16 (10.78 ± 3.37)	2–17 (11.71 ± 3.51)	0.412
Gender			0.781
Male	14	19	
Female	9	16	
Symptom duration (months, mean ± SD)	2–89 (24.61 ± 21.01)	1–244 (26.86 ± 43.29)	0.446
Initial dose of AZA (mg/kg/d)	0.29–1.32 (0.79 ± 0.28)	0.43–1.61 (0.80 ± 0.29)	0.718
Maximum dose of AZA (mg/kg/d)	0.74–1.96 (1.34 ± 0.34)	0.70–1.81 (1.22 ± 0.32)	0.047
WBC change following AZA (*10^9^/L)	−8.93∼-0.40 (−3.21 ± 2.52)	−13.24–1.32 (-2.85 ± 3.35)	0.677
HGB change following AZA (g/L)	−36.00–36.00 (−0.06 ± 16.67)	−38.00–18.00 (-1.43 ± 11.80)	0.734
PLT change following AZA (*10^9^/L)	−179.00 ± 65.00 (−38.44 ± 77.76)	−308.00–225.00 (-32.43 ± 96.75)	0.828
Concomitant therapy			
Corticosteroids (number)	5	7	1.000
Mesalazine (number)	17	26	0.749
Biologics (number)	17	29	1.000

## 4 Discussion

Despite the ongoing development of new drugs such as biologics and small molecule medications, thiopurine drugs still play a significant role in the treatment of IBD. The early introduction of thiopurines during the maintenance phase of IBD enables 73% of patients to achieve at least 1 year of hormone-free remission. Following 1 year of azathioprine treatment, 76.9% of children with UC and 47.6% with CD can achieve endoscopic remission (Giugliano et al., 2018). However, thiopurines are related to adverse reactions including gastrointestinal intolerance, influenza-like symptoms, pancreatitis, hepatitis, rash, myelosuppression, and infections secondary to neutropenia. Among these adverse effects, myelosuppression is the most prevalent and has the potential to lead to life-threatening infections. This study investigated the importance of pharmacogenetic testing prior to the use of azathioprine in Chinese pediatric IBD and analyzed the adverse reactions that occurred following azathioprine administration, finding that pharmacogenetic testing was beneficial in guiding clinical treatment and to some extent predict the likelihood of subsequent myelosuppression.

Leukopenia is the most common manifestation of myelosuppression resulting from thiopurine medications, and it can develop at any point after the commencement of treatment. It may also arise abruptly without any symptoms or warning signs. Thirty-seven percent of patients experience leukopenia within 3 months, 22.4% within 3–6 months, 13.8% within 6–12 months, 22.4% within 12–24 months, and 14.7% after 24 months (Ribeiro et al., 2023). Similarly, in our study, the median onset of myelosuppression was at 300 days following the initiation of azathioprine, with 30.4% occurring within the first 60 days and 69.6% after 60 days. Nonetheless, it should be noted that most patients in our study were pharmacogenetically normal and that myelosuppression can also manifest several years post-medication, underscoring the necessity for ongoing monitoring of peripheral blood parameters even with normal pharmacogenetic results.

The metabolites of azathioprine primarily include 6-mercaptopurine, thiouracil, 6-TGN, and 6-methylmercaptopurine (6-MMP). TPMT is a pivotal enzyme involved in the metabolism of thiopurines, and its genetic polymorphisms are linked to reduced enzyme activity, which in turn increases the risk of myelosuppression induced by thiopurines (Ribeiro et al., 2023). The four genotypes TPMT*2, TPMT*3A, TPMT*3B, and TPMT*3C account for over 95% of *TPMT* gene variants, yet there are notable differences in the genetic polymorphisms of pharmacogenomics among various populations (Kennedy et al., 2024). The most common TPMT*3A genotype is prevalent among Caucasians (3.2%–5.7%), whereas the relatively common *TPMT* variant in Africa and Asia is the TPMT*3C genotype (0.5%–1.5%) (Ribeiro et al., 2023). In this study, the mutation probability of three loci in the *TPMT* gene of 227 IBD children was 3.1%, with all of them occurring in TPMT*3C, indicating that the incidence of *TPMT* variant loci can vary significantly among different regions, and TPMT*3C is more significant than TPMT*2/*3B for Chinese patients.

For Caucasians, *TPMT* plays a crucial role in predicting leukopenia caused by thiopurines, but its predictive value is limited in Asian populations. NUDT15 is another gene that significantly influences the metabolism of thiopurine drugs. Its protein product can dephosphorylate 6-TGN, thereby preventing the incorporation of metabolites into DNA or RNA. When mutations occur in the NUDT15 gene, the enzymatic activity of NUDT15 is reduced, leading to an increased risk of adverse effects from thiopurine therapy (Khaeso et al., 2021). Among the most common genetic polymorphisms of NUDT15, the C415T variant is highly prevalent in East Asian populations but rare in Caucasians. Furthermore, compared to *TPMT*, the *NUDT15* C415T variant is associated with an increased susceptibility to early and severe myelosuppression following thiopurine treatment, making it a superior predictor of thiopurine-related myelosuppression in Asian populations (Yang et al., 2014; Relling et al., 2019; Banerjee et al., 2020; Chao et al., 2021; Kennedy et al., 2024). Moreover, the same genetic variant can yield inconsistent results across different ethnic groups. While *NUDT15* mutations are more prone to cause early severe myelosuppression in Asian populations, patients with this variant in the United Kingdom do not exhibit an elevated risk of toxicity after taking thiopurines, highlighting the significance of *NUDT15* genotype testing in Asian populations (Chao et al., 2017; Desai et al., 2023; Coelho et al., 2024). In this study, the prevalence of *NUDT15* mutations was notably higher than that of *TPMT*, with an overall mutation rate of 26.4% for *NUDT15* C415T, compared to a mutation rate of 3.1% for 3 TPMT loci, indicating that *NUDT15* is a crucial component in pharmacogenetics for thiopurines in the Chinese population. In this study, all three pediatric patients with intermediate NUDT15 metabolic activity developed myelosuppression after receiving azathioprine, further underscoring the need for cautious use of thiopurines in Chinese children with IBD who carry the NUDT15 C415T variant. However, the sample size of patients with pharmacogenomic abnormalities treated with azathioprine in this study was limited. Therefore, the generalizability of these findings requires validation through larger-scale prospective studies.

Other genes, including *ITPA*, *GST*, *AOX1*, *DHFR*, and *FTO*, may also influence the metabolism of thiopurines (Kim et al., 2017; Casajús et al., 2022; Dickson et al., 2022; Coelho et al., 2024). For IBD patients treated with thiopurines, drug dosage, baseline WBC count, and the choice of mercaptopurine were also identified as independent risk factors for leucopenia (Broekman et al., 2017). In our study, 36.4% (20/55) of patients who experienced myelosuppression had normal pharmacogenomics, indicating that genotypes cannot solely account for phenotypic drug responses, and that phenotypes themselves cannot be completely replaced by genotypes. A recent prospective study in China on CD demonstrated that among patients with normal TPMT/NUDT15 activity, 15.6% (17/109) experienced late leukopenia, and in those with a moderate decline in TPMT/NUDT15 activity the incidence of late leukopenia was 64.1% (25/39). It is important to note that not all adverse reactions associated with thiopurines can be explained by the commonly studied thiopurine metabolites such as 6-MMP and 6-TGN, and thus more appropriate phenotypic marker is required to predict drug toxicity (Sheffield et al., 2009; Luo et al., 2022; Yang et al., 2024). As a thiopurine metabolite associated with NUDT15, 6-diazo-5-oxo-L-norleucine (DNATG) is proved to be superior to 6-TGN in predicting whether TPMT/NUDT15-normal patients will develop late leukopenia. When the DNATG cutoff value is set at 315.72 fmol/μg DNA, its sensitivity in predicting late leukopenia is 88% and its specificity is 85%, suggesting that detecting DNATG at the onset of medication can provide a better prediction of late leukopenia (Zhu et al., 2022; Yang et al., 2024). The role of metabolites mentioned above in pediatric IBD in China needs further investigation.

Concomitant medications, such as allopurinol, can also influence thiopurine metabolism. Regarding the combination with 5-aminosalicylic acid (5-ASA), the evidence on its impact on thiopurine metabolism and toxicity remains inconsistent. Researches have indicated that 5-ASA can elevate the levels of the active metabolite 6-TGN of azathioprine, decrease the ratio of 6-MMP/6-TGN, and thereby increase the risk of leukopenia (Lee et al., 2015). The interaction between these drugs is not solely dependent on drug dosage but also on drug formulation (Hande et al., 2006; de Boer et al., 2007; de Graaf et al., 2010). When a high dose of mesalazine is necessary in clinical practice, it is crucial to meticulously assess the adverse reactions associated with the combination of thiopurines (Ribeiro et al., 2023). However, some studies have reported contradictory findings, showing no increased risk of leukopenia or elevated 6-TGN levels with the co-administration of 5-ASA and thiopurines (Broekman et al., 2017). In this study, consistent with the latter, there was no statistically significant difference in the concomitant mesalazine between the myelosuppression group and the non-myelosuppression group, which may be attributed to the relatively low drug dosage used. Further research is needed in China to investigate the impact of the combination of these two drugs on myelosuppression in pediatric IBD.

Pharmacogenomics plays a crucial role in guiding the use of thiopurines, and economic analyses have demonstrated overall benefits for patients, supporting the current recommendation to conduct pharmacogenomic testing prior to initiating thiopurine therapy (Zeng et al., 2021; Desai et al., 2023; Valdez-Acosta et al., 2023). Clinical guidelines generally suggested a dosage of 2.0–2.5 mg/kg/day for azathioprine in IBD with normal metabolic activity, while the Clinical Pharmacogenomics Implementation Consortium recommended a dosage of 2.0–3.0 mg/kg/day (Relling et al., 2019; van Rheenen et al., 2020; Lee et al., 2023). It is important to note, however, that the recommended doses in these guidelines are primarily based on clinical studies of Caucasian populations. For other regions and ethnic groups, including Asian populations with varying genotypes and a different risk of myelosuppression compared to Caucasian populations, further assessment is required for the dosing, therapeutic effects, and toxic reactions of azathioprine. Studies in Japanese populations have indicated that lower doses of azathioprine are sufficient to achieve adequate levels of active metabolites and clinical efficacy (Andoh et al., 2008; Komiyama et al., 2008; Ohtsuka et al., 2010). In a study by Kim et al. involving 286 Korean patients, the dosage of azathioprine was 1.84 ± 0.54 mg/kg/day, with only 35.3% of patients able to tolerate a dosage of 2.0 mg/kg/day or more (Kim et al., 2010). Chao et al. then investigated the optimal treatment strategy based on the *NUDT15* C415T genotypes in Chinese CD patients in a randomized controlled trial. The CC genotype received the standard dose, the CT genotype received 50% of the standard dose, and the TT genotype received alternative medications. The results revealed that the above optimized strategy based on *NUDT15* C415T could effectively reduce early leukopenia in Chinese CD patients using thiopurines without compromising efficacy, but it did not prevent late leukopenia (Chao et al., 2021). In our study, the maximum dose of azathioprine for children in the myelosuppression group and the non-myelosuppression group was 0.74–1.96 mg/kg/day (1.34 ± 0.34) and 0.70–1.81 mg/kg/day (1.22 ± 0.32), respectively, which was also lower than the recommended dosage in the guidelines. In summary, further exploration is necessary regarding the optimal treatment dose, toxic reactions, and treatment regimen adjustments for azathioprine in Asian children with IBD.

This study has several limitations that must be acknowledged. First, the retrospective nature of this study introduces potential selection bias and incomplete data collection, which may compromise the generalizability of the findings. Second, the limited sample size reduces the statistical power of the analysis, potentially resulting in an inability to identify significant associations or differences that may exist in a broader population. Thirdly, the single-center design may limit the external validity and generalizability of the results. Furthermore, the pharmacogenomic testing conducted in this study did not cover all known thiopurine drug metabolism genes, which may cause pharmacogenetic results partially omissive. Lastly, the metabolites of azathioprine such as 6-TGN, DNATG, and 6-MMP were not involved in this study, so the relationship between the occurrence of myelosuppression and the phenotype could not be determined. Future prospective, multicenter studies with larger cohorts are warranted to validate our findings and establish more robust evidence.

## 5 Conclusion

Pharmacogenomic testing is of significant importance for guiding the use of thiopurines and predicting the risk of myelosuppression. Testing for *TPMT* and *NUDT15*, especially *NUDT15*, is recommended before the administration of thiopurines in Chinese population. Treatment based on proactive pharmacogenomics still demands vigilant monitoring of adverse reactions, even when pharmacogenomic results are normal.

## Data Availability

The raw data supporting the conclusions of this article will be made available by the authors, without undue reservation.
